# Positive parental history of diabetes is associated with early diagnosis, better dietary compliance, and glycemic control among type 2 diabetes patients in southern Sri Lanka

**DOI:** 10.1186/s13098-024-01394-w

**Published:** 2024-07-11

**Authors:** Warsha De Zoysa, Thilak Priyantha Weerarathna, Keddagoda Gamage Piyumi Wasana, Miyuru Kavinda Weerarathna, Vidarsha Senadeera

**Affiliations:** 1 Department of Medicine, Faculty of Medicine, University of Ruhuna, Galle, Sri Lanka; 2 Diabetes Centre, Cooperative Hospital, Galle, Sri Lanka; 3 University Medical Unit, Teaching Hospital, Karapitiya, Galle, Sri Lanka

**Keywords:** Age at diagnosis, Dietary compliance, Gestational diabetes mellitus, Glycemic control, Parental history, Type 2 diabetes mellitus

## Abstract

**Background:**

Parental history of diabetes is an established risk factor for type 2 diabetes mellitus (T2DM). There is limited data on the association of parental history with the prevalence of T2DM in Sri Lanka. The study aims to examine the prevalence and correlation of parental history and factors such as the onset age, glycaemic control, and self-reported dietary compliance among T2DM patients. With a rising incidence of T2DM in Sri Lanka, understanding the impact of parental history on age at diagnosis and glycemic control can aid in targeted screening and interventions.

**Methods:**

A cross-sectional study was carried out on 500 T2DM patients attending a diabetes clinic in Galle, the capital of Southern Sri Lanka with a multiethnic population. Convenient sampling strategy was followed in the recruitment process and a questionnaire-based method was used to collect the data. All the collected data was analysed using SPSS V 25.0.

**Results:**

51.2% had a parental history of T2DM, and those with a positive parental history were diagnosed six years earlier than those with a negative parental history (*p* < 0.001). A significant correlation between parental history and gestational diabetes mellitus (GDM) was observed (*p* < 0.001). Patients with a parental history reported better dietary adherence (*p* < 0.001). Binary logistic regression analysis revealed patients with positive parental history had significantly lower HbA_1C_ (*p* = 0.003, OR = 0.748).

**Conclusion:**

T2DM patients with a parental history showed significant association with early diabetes onset, GDM, better glycemic control, and dietary adherence.

## Background

Type 2 diabetes mellitus (T2DM) has become a crucial health concern worldwide having a significant impact on human life and healthcare costs. Having been diagnosed with diabetes has a negative impact on one’s functional capabilities and quality of life thereby leading to significant morbidity and premature deaths [1]. In 2000, it was evaluated that diabetes affected approximately 171 million people worldwide; by 2011, this number had grown to more than 366 million, and the numbers are expected to surpass 552 million by the year 2030, reflecting a continuous increase across all regions around the globe [2]. In 2017, diabetes alone accounted for more than 10 lakh deaths every year, making it one of the top 10 diseases leading to mortality. This increase is alarming compared with that in 1990 when T2DM was ranked as the 18th prime cause of death [1]. The recent literature reveals that individuals younger than 60 years account for more than one-third of deaths related to diabetes [1].

The implication of diabetes mellitus, both in terms of prevalence and adult numbers with T2DM, has significantly become higher in developing nations in contrast to developed countries [3, 4]. Approximately 75% of people with diabetes live in developing nations, with the vast majority believed to remain undiagnosed [5]. T2DM is a widespread epidemic in Asia characterized by a speedy increase in shorter periods and emergence at a relatively early age among individuals with lower body mass indices as compared to those in developed countries [6]. By 2030, the number of adult diabetes cases are expected to be as high as 113 million and by 2045 it will be almost 151 million in Southeast Asia [7]. A recent meta-analysis of 1.7 million adults found that the prevalence of diabetes increased in both rural and urban India from 2.4% to 3.3% in 1972 to 15.0% and 19.0%, respectively, in 2015-2019 [5]. A meta-analysis in 2016 analysing 77 Chinese studies revealed that the prevalence rates of T2DM as 11.4% in urban China and 8.2% in rural China [8]. Sri Lanka is a low- and middle-income country with twenty-one million individuals in South Asia that is facing substantial transformations such as ageing, obesity, urbanization, and decreased opportunities for exercise, which all contribute to a growing epidemic of diabetes [9]. According to Rannan-Eliya et al. (2019), 23.0% of adults in Sri Lanka have diabetes, making it the country with the highest prevalence of this disease in South Asia [7]. Furthermore, the same study showed that despite reachable access to healthcare services, greater than one-third of Sri Lankan adults suffering from diabetes remained undiagnosed. A meta-analysis in 2022 revealed that the pooled T2DM prevalence in Sri Lanka is the greatest in the most recent era of 2011–2021 (17.25%) than in the 2000s (11.84%) and 1990s (5.62%), showing an alarming growth over the last three decades [10]. Moreover, the same meta-analysis showed that nearly three out of every four people with T2DM are unaware they have the illness [10].

A variety of genetic, behavioral, demographic, and psychosocial factors have been implicated in the etiology of T2DM. Genetic predispositions to T2DM have been known for a long time and are the focus of related research in different settings [11]. Scott R et al. found that family history of T2DM remains a strong and independent risk factor even after adjusting for established risk factors such as unfavorable anthropometric and lifestyle variables [12]. Studies amongst various ethnic groups have reported a relation between positive family history and the risk of diabetes which increases by two- to sixfolds [13]. In 2017, Viner et al. reported that 60% of mothers with a younger onset had either of their family and 30% had either of their grand family suffering from diabetes [14]. An Indian study carried out on the population with a family history inferred that the average age of onset of T2DM was 55.95 years in the first generation and 38.4 years in the second generation respectively [15]. Diabetes and family history had a graded association in the Sri Lankan population, because the incidence increased with the growing number of generations afflicted, and the prevalence was highest when family history was positive for both parents (32.9%).^16^ Another Sri Lankan study indicated that individuals with a positive family history were 3.5 times more likely to get the disease [16].

Although there is ample evidence for the familial association and T2DM in other parts of the world, there is a relative scarcity of Sri Lankan data on this topic. Sri Lankan studies on the association between parental history, disease onset, and metabolic control were not available in the literature. With an increasing prevalence of T2DM and undiagnosed cases in Sri Lanka, understanding the impact of parental history on age at diagnosis and glycemic control would allow targeted screening and interventions. Therefore, our study aimed to study the prevalence of parental history (either maternal or paternal and both parents) and prospect the associations of parental history with age at diagnosis of diabetes, gestational diabetes mellitus (GDM), intensity of glucose control, and self-reported dietary and drug compliance among patients with T2DM in Sri Lanka.

## Methods

### Study design and setting

The study was cross-sectionally designed and conducted among previously diagnosed patients with T2DM attending an Outpatient Diabetes Clinic in southern Sri Lanka between August 2022 and January 2023. STROBE checklist for cross-sectional studies was considered in designing the experimental protocol of the study. The Outpatient Diabetes Clinic, at Cooperative Hospital, Galle was held on three days per week having 1020 registered number of T2DM patients during the study time. Generally, patients can establish care at the Clinic either through external referral or self-referral.

The necessary Ethics Committee approval was obtained from the Institute. Written informed consents were also obtained from each patient before the study commencement.

### Sample size and patients

The sample size was determined with the prevalence of T2DM in Sri Lanka as 23% and the margin of error of 3.8% [7].

n = Z^2^P (1-P) / W^2^

where, n = minimum sample size, Z = 1.96 (for 95% confidence interval). The calculated minimum sample size was 471, hence the final sample size was kept at 500 to make allowance for missing data. A total number of 500 patients with T2DM who were aged more than 18 years were enrolled for the study. Patients with T1DM, newly diagnosed T2DM patients of less than three months, with viral hepatitis-like illness in the past, with cirrhosis or who were taking medications for any chronic liver disease, with a self-reported history of alcohol intake, or who were pregnant or lactating fell into the exclusion criteria of the study. Considering these inclusion and exclusion criteria, every consecutive subject (regardless of gender) in the Diabetes clinic Register was recruited until the required sample size was achieved.

### Data collection

The data, including demographic and disease-related data, were collected using interviewer-administered questionnaires. Each study participant was directly questioned if any of their parents (alive or deceased) had their diabetes diagnosed by any medical doctor. It was enquired from the female participants whether they had been diagnosed with GDM during any of their pregnancies. Patients with maternal diabetes, paternal diabetes, or both maternal and paternal diabetes were considered separately. Self-reported dietary and drug compliance was checked using a visual analogue scale ranging from 0 to 10 [17, 18].

Both systolic and diastolic blood pressures (SBP/DBP) were recorded and the mean of three consecutive measurements were taken five minutes after the patients were in a sitting position. SBP ≥ 140 mmHg and DBP ≥ 90 mmHg were termed as hypertensive.

The enrolled patients were subjected to biochemical investigations, which included measuring glycosylated hemoglobin (HbA_1C_), high-density lipoprotein cholesterol (HDL-C) levels, total cholesterol (TC), and triglyceride (TG). Assessment of HbA_1C_ was done using high-performance liquid chromatography having a fully automated analyser (BIORAD D analyser, USA). Lipid profile parameters were estimated on a fully automated analyser based on spectrophotometric principles (Huma star − 600 HS- Germany). The Friede-Wald equation was used for calculating low-density lipoprotein cholesterol (LDL-C). An identification number was given to each study subject at the time of collection of their data. All the data collected during the study were stored on one of the investigators’ laptops and in a data repository with respect to their identification numbers. The data were not shared with persons other than the investigators of the study, and all the investigators had access to the data repository.

### Statistical analysis

Descriptive statistics were applied for the data set in terms of mean ± standard deviation (SD) and frequency (percentages). The selected characteristics: continuous data, between the study subjects with parental history and without the parental history were compared using student’s unpaired t-test. Categorical data were compared using the chi-square test. Association of age, age at disease onset, dietary compliance, and HbA_1C_ with different groups according to parental history; maternal diabetes, paternal diabetes, or both maternal and paternal diabetes, was assessed using One-way ANOVA followed by Tukey’s test. Association between parental history and the selected parameters was assessed via binary logistic regression model in terms of odds ratios (ORs) with 95% confidence intervals (CIs). In the model, age, age at onset of T2DM, dietary compliance, and HbA_1C_ were independent variables whereas the presence or absence of parental history was the dependent variable. SPSS version 25.0 conducted the statistical analysis, with a significance threshold set at *p* ≤ 0.05.

## Results

The average age of the entire study sample (*n* = 500) was 60 ± 11 years, and females constituted 60.6% (*n* = 303) of the participants. A greater proportion of patients had good self-reported dietary and drug compliance (self-reported score 7 or above), at 37% and 92%, respectively. Overall, 67.2% of the patients were receiving drug treatment for hypertension, and 82.2% were taking medication for dyslipidemia. All these basic characteristics are stated in Table 1.

**Table 1:** Baseline characteristics of the study subjects

Characteristic	Total (*n* = 500)
Gender (%) of women	303 (60.6)
Mean ± SD age (years)	60 ± 11
Mean ± SD age at onset of T2DM (years)	49 ± 11
Dietary compliance	
Number (%) of good (score ≥ 7)	185 (37.0)
Number (%) of moderate (score 6 − 3)	183 (36.6)
Number (%) of poor (score ≤ 2)	132 (26.4)
Drug compliance	
Number (%) of good (score ≥ 7)	460 (92.0)
Number (%) of moderate (score 6 − 3)	36 (7.2)
Number (%) of poor (score ≤ 2)	4 (0.8)
Number (%) with hypertension	336 (67.2)
Number (%) with dyslipidemia	411 (82.2)
Mean ± SD HbA_1C_ (%)	7.5 ± 1.0

HbA_1C_; glycated hemoglobin, T2DM; type 2 diabetes mellitus

Of the studied patients, A family history of T2DM was observed in 51.2%, with 31% from the maternal side, 12% from the paternal side, and 8% having both parents affected. Importantly, those with a family history of diabetes were diagnosed with T2DM at an earlier age (Table 2). T2DM patients with a positive parental history demonstrated better glycaemic control, as evidenced by lower HbA_1C_ levels, than those without such a history.

**Table 2:** Characteristics of the patients with and without parental history

Characteristic	Study subjects with parental history (*n* = 256)	Study subjects without parental history (*n* = 244)	*p*-value
Gender (%) of women	165 (64.5)	138 (56.6)	0.549
Mean ± SD age (years)	57 ± 11	64 ± 10	< 0.001
Mean ± SD age at onset of T2DM (years)	46 ± 11	52 ± 10	< 0.001
Dietary compliance			< 0.001
Number (%) of good (score ≥ 7)	96 (37.5)	89 (36.5)	
Number (%) of moderate (score 6 − 3)	88 (34.4)	95 (38.9)	
Number (%) of poor (score ≤ 2)	72 (28.1)	60 (24.6)	
Drug compliance			0.599
Number (%) of good (score ≥ 7)	235 (91.8)	225 (92.2)	
Number (%) of moderate (score 6 − 3)	19 (7.4)	17 (7.0)	
Number (%) of poor (score ≤ 2)	2 (0.8)	2 (0.8)	
Number (%) with hypertension	165 (64.5)	171 (66.8)	0.675
Number (%) with dyslipidemia	215 (84.0)	196 (76.6)	0.144
Mean ± SD HbA_1C_ (%)	7.4 ± 1.0	7.6 ± 1.0	0.042

HbA_1C_; glycated hemoglobin, T2DM; type 2 diabetes mellitus

Amongst the 303 females, 261 have undergone pregnancy and 248 females had children. Of all females, 51 (16.8%) had GDM, and among the females with GDM, 35 (68.6%) had a positive parental history, including significant higher cases of maternal (56.9%) than paternal (7.8%) or both maternal and paternal (3.9%). (*X*^2^ = 12.360, *p* = 0.006).

The results of ANOVA revealed that there was a difference in the mean age at disease onset between the different groups according to parental history (F (2,253) = 5.790, *p* = 0.003) which was statistically significant. According to the post hoc analysis, the group of patients with both parental history of diabetes had a significantly lower mean age at diagnosis (41 ± 12 years) than did the T2DM patients with maternal diabetes (47 ± 11 years) or paternal diabetes (48 ± 10 years). However, age, dietary compliance, and HbA_1C_ did not significantly differ between the groups of patients with maternal diabetes, paternal diabetes, or both maternal and paternal diabetes.

The binary logistic regression model explained 14.2% (Nagelkerke *R*^2^) of the positive parental history of T2DM patients and correctly classified 64.0% of the patients. Age and HbA_1C_ were significantly and positively associated with active parental history in patients with T2DM (Table 3).

**Table 3:** Factors associated with patients with a positive parental history

Variable	OR	95% CI	*p*-value
Age (years)	0.956	0.932–0.981	0.001
Age at onset of T2DM (years)	0.980	0.955–1.007	0.140
Dietary compliance			
Poor (score ≤ 2)	0.876	0.536–1.1432	0.597
Moderate (score 6 − 3)	0.739	0.477–1.145	0.175
HbA_1C_ (%)	0.748	0.619–0.904	0.003

HbA_1C_; glycated hemoglobin, T2DM; type 2 diabetes mellitus

## Discussion

This cross-sectional study involving 500 patients with T2DM revealed several important findings on the genetic associations of the rapidly escalating diabetes epidemic. All the other T2DM patients (51%) had a parental history, the maternal being the most common (31%), followed by paternal history (12%), and both parents with T2DM (8%). Moreover, those with a parental history of diabetes were diagnosed with T2DM six years younger than those without a parental history (46 ± 11 years vs. 52 ± 10 years, *p* = 0.05). Of the 303 females included in this study, 51 (16.8%) had a history of GDM. A correlation was observed between GDM history and T2DM positive parental history (*p* = 0.006) which was significant. Among females with diabetes, a significantly greater percentage (56.9%) had a positive maternal history than had a positive paternal history (7.8%). Of the 51 females with a history of GDM, only four had diabetes. We also found that those with a positive parental history had significantly better glucose control, as measured by HbA_1C_ and self-reported dietary compliance.

A strong link between T2DM and parental history (51%) was supported by several previous studies [19–23]. According to an Indian study, 52.5% of patients with T2DM had a parental history [19]. Like this study, many previous studies have shown a strong relationship between diabetes in mother and offspring [19, 22–25]. According to Papazafiropoulou et al. in Greece, the prevalence of maternal diabetes was threefold greater compared to fathers of patients with T2DM [24]. As reported by Evuru et al. in India, 23.2% of patients had a diabetic mother, 19.4% had a paternal, and 5.6% had both diabetic [19]. A study performed by Crispim et al. in Brazil showed that diabetes in mothers was approximately twice as common as diabetes in fathers [22]. These studies revealed that a maternal history of T2DM was more prevalent among individuals diagnosed with T2DM than among those with a paternal history.

The group having a parental diabetic history was diagnosed with T2DM six years younger than the group with no history. Early age at diagnosis of T2DM among those with a parental history of diabetes has been reported in previous studies conducted in other countries [11, 20, 24–26]. Bruce et al. showed equivalent results in which patients with a parental history of diabetes developed T2DM five years earlier than their counterparts having no parental history [20]. Additionally, the conclusions of this study indicate that individuals with both parental history of diabetes exhibited a significantly lower mean age of onset of T2DM (41 ± 12 years) than individuals with only maternal diabetes (47 ± 11 years) or paternal diabetes (48 ± 10 years). A Chinese study revealed that parental T2DM was associated with early-onset disease, and the ORs were 2.866 for both parents with T2DM, 2.738 for paternal T2DM, and 1.536 for maternal T2DM [24]. According to the literature, a family history is linked with the earliest onset of the disease, followed by a paternal history [20, 24]. However, there was no correlation between diabetes incidence or history of paternal T2DM compared to maternal T2DM.

In parallel with the increase in T2DM incidence, the incidence of GDM is seen to be increasing in the population [26]. According to a systematic review, one in every ten pregnant women in Eastern and Southeast Asia had GDM [27]. In this study, it was observed that there was a substantial connection between family history and GDM. Several previous studies have shown that a familial occurrence of diabetes among primary relatives is a risk factor for GDM. The risk is highest when the family history is positive for both parents [26, 28, 29]. However, the significantly high incidence of maternal history of T2DM in patients with GDM observed in this study is not consistent with the literature and is currently a subject of debate [26].

Patients with a positive parental history in this study had better self-reported dietary compliance and lower HbA_1C_. No significant differences were observed in diabetes compliance or metabolic control among individuals with a positive parental history of T2DM when subgroup analysis was conducted based on the type of parental T2DM. The better dietary compliance and glycemic control of patients with a positive parental history in this study may be attributed to their similar living environment, as well as being more vigilant and aware of diabetes, resulting in better adherence and control. The same living environment and shared expectations of both parents and children may accelerate parental efforts to improve T2DM incidence and patient adherence to dietary interventions to improve glycemic control. In contrast, many previous studies have shown a negative association between family history and glycemic control [20, 30, 31]. However, Cheung et al. reported similar findings to those of this study, with 86,931 patients from 11 Asian countries reporting lower HbA_1C_ among patients with a positive parental history as compared to those with a negative parental history [32].

Enrolling every consecutive subject (regardless of gender) in the Diabetes clinic Register was practiced in the present study and therefore it mitigated the sampling bias. Even though there are previous studies from Sri Lanka on family history of diabetes, in this study, we went beyond that to find out the association, which is clinically relevant for the management of patients. We believe our findings of early onset of T2DM among patients with parental history would enable clinicians to subject such patients to early screening. As described in the recent meta-analysis, 75% of previously undiagnosed diabetes in Sri Lanka can be mitigated by early screening of people with positive parental histories. Healthcare systems must be improved to ensure easy access to screening services for high-risk people. At the community level, appropriate and intensive educational programs for high-risk individuals with a positive parental history should be organized. Furthermore, a parental history of T2DM is a dominant risk factor for developing GDM. A parental history of T2DM should be taken as a strong risk factor, and appropriate measures should be taken during the preconception period and early pregnancy to minimize fetal and maternal complications due to GDM.

Considering its limitations, the findings of this study must be considered. The female patient percentage in the group accounted for 60.6%, which could cause a result bias. Secondly, details of the family history of diabetes were collected based on patient memories about their family history. Drug and dietary compliance were assessed using a visual analogue scale, which is not a validated tool. The study was conducted at a single center, and the participants were predominantly from urban areas in southern Sri Lanka. Therefore, generalizability of the present study findings for all Sri Lankans is questionable. Future studies on T2DM patients who represent several localities around the country are warranted.

## Conclusions

The current study centers on the notable impact of family history on the rising global occurrence, onset of age, self-reported dietary adherence, and long-term glucose control in patients with T2DM in a South Asian country amid the pandemic. These findings will be useful for screening and managing patients with diabetes. The significant association between maternal history of T2DM and GDM observed in this study warrants additional research since this association is currently under debate. Furthermore, we suggest maintaining a register in maternity care centers of females with GDM and following up on their offspring with the aim of early screening for T2DM as a potential way to detect the disease early.

## Data Availability

No datasets were generated or analysed during the current study.

## Abbreviations

DBP: Diastolic blood pressure
GDM: Gestational diabetes mellitus
HbA1c: Glycosylated hemoglobin
HDL: C-high-density lipoprotein cholesterol
OR: Odds ratio
SBP: Systolic blood pressure
TC: Total cholesterol
TG: Triglyceride
T2DM: Type 2 diabetes mellitus

## References

[1] Abdul M, Khan B, Hashim MJ (2020). Epidemiology of type 2 diabetes – global burden of disease and forecasted trends. J Epidemiol Glob Health.

[2] Alotaibi A, Perry L, Gholizadeh L, Al-Ganmi A (2017). Incidence and prevalence rates of diabetes mellitus in Saudi Arabia: an overview. J Epidemiol Glob Health.

[3] Guariguata L, Whiting DR, Hambleton I, Beagley J, Linnenkamp U, Shaw JE (2014). Global estimates of diabetes prevalence for 2013 and projections for 2035. Diabetes Res Clin Pract.

[4] Zhou B, Lu Y, Hajifathalian K (2016). Worldwide trends in diabetes since 1980: a pooled analysis of 751 population-based studies with 4.4 million participants. Lancet.

[5] Priyanga Ranasinghe (2021). Others, ‘Prevalence and trends of the diabetes epidemic in Urban and Rural India: a pooled systematic review and Meta-analysis of 1.7 million adults’. Ann Epidemiol.

[6] Ma H, Gong Y, Liu Y (2011). Prevalence of diabetes and prediabetes mellitus in the first-degree relatives of patients with type 2 diabetes in Chengdu. Sichuan Da Xue Xue bao Yi xue ban.

[7] Rannan-Eliya RP, Wijemunige N, Perera P (2023). Prevalence of diabetes and prediabetes in Sri Lanka: a new global hotspot-estimates from the Sri Lanka Health and Ageing Survey 2018/2019. BMJ Open Diabetes Res Care.

[8] Yang L, Shao J, Bian Y (2016). Prevalence of type 2 diabetes mellitus among inland residents in China (2000–2014): a meta-analysis. J Diabetes Investig.

[9] Pruthu Thekkur and others. Status and challenges with Human resources, Information Systems, Drugs and Laboratory Services. Healthc (Basel Switzerland). 2022;10(11). 10.3390/healthcare10112251 10.3390/healthcare10112251 PMC9691080 36360593

[10] Sohail Akhtar and others. Prevalence of type 2 diabetes and pre-diabetes in Sri Lanka: a systematic review and Meta-analysis. BMJ Open. 2023;13. 10.1136/bmjopen-2022-068445 10.1136/bmjopen-2022-068445 PMC10462943 37640460

[11] Jeong SU, Kang DG, Lee DH (2010). Clinical characteristics of type 2 diabetes patients according to family history of diabetes. Korean Diabetes J.

[12] Scott RA. and Others, ‘The link between family history and risk of type 2 diabetes is not explained by Anthropometric, Lifestyle or genetic risk factors: the EPIC-InterAct study.’, Diabetologia, 56.1 (2013), 60–910.1007/s00125-012-2715-x 10.1007/s00125-012-2715-x PMC4038917 23052052

[13] Ma RCW, Chan JCN (2013). Type 2 diabetes in East asians: similarities and differences with populations in Europe and the United States. Ann N Y Acad Sci.

[14] Viner R, White B, Christie D (2017). Type 2 diabetes in adolescents: a severe phenotype posing major clinical challenges and public health burden. Lancet.

[15] Panikar VK, Joshi SR, Kakraniya P, Nasikkar N, Santavana C (2008). Intergeneration comparison of type-2 diabetes in 73 Indian families. J Assoc Physicians India.

[16] Amarasinghe S, Balakumar S, Arasaratnam V (2015). Prevalence and risk factors of diabetes Mellitus among adults in Jaffna District. Ceylon Med J.

[17] Thilak Priyantha Weerarathna and others. ‘Association of Self-Reported Dietary and Drug Compliance with Optimal Metabolic Control in Patients with Type 2 Diabetes: Clinic-Based Single-Center Study in a Developing Country’, ed. by José María Huerta, Journal of Nutrition and Metabolism, 2018 (2018), 10.1155/2018/3421476 10.1155/2018/3421476 PMC6081544 30140455

[18] Annette Winkler and others (2002). Monitoring adherence to prescribed medication in type 2 Diabetic patients treated with sulfonylureas. Swiss Med Wkly.

[19] Geetha A, Gopalakrishnan S, Umadevi R (2017). Study on the impact of family history of diabetes among type 2 diabetes mellitus patients in an urban area of Kancheepuram district, Tamil Nadu. Int J Community Med Public Heal.

[20] Bruce DG, Van Minnen K, Davis WA (2010). Maternal family history of diabetes is associated with a reduced risk of cardiovascular disease in women with type 2 diabetes: the fremantle diabetes study. Diabetes Care.

[21] Crispim D, Canani LH, Gross JL, Tschiedel B, Souto KEP, Roisenberg I (2006). Familial history of type 2 diabetes in patients from southern Brazil and its influence on the clinical characteristics of this disease. Arq Bras Endocrinol Metabol.

[22] Al-Harbi EM, Farid EM, Darwish AH, Gumaa KA, Giha HA (2016). Women transmits type 2 diabetes mellitus (T2DM) more than men: evidence from parental inheritance of T2DM among Bahrainis. Exp Clin Endocrinol Diabetes.

[23] Papazafiropoulou A, Sotiropoulos A, Skliros E (2009). Familial history of diabetes and clinical characteristics in Greek subjects with type 2 diabetes. BMC Endocr Disord.

[24] Hao Z, Huang X, Liu X, He F, Shao H (2022). Association analysis between different diabetic family history and gender with diagnosed age of type 2 diabetes mellitus: a cross-sectional study in Tianjin, China. Inquiry.

[25] Svensson E, Berencsi K, Sander S (2016). Association of parental history of type 2 diabetes with age, lifestyle, anthropometric factors, and clinical severity at type 2 diabetes diagnosis: results from the DD2 study. Diabetes Metab Res Rev.

[26] Monod C, Kotzaeridi G, Linder T (2023). Prevalence of gestational diabetes mellitus in women with a family history of type 2 diabetes in first- and second-degree relatives. Acta Diabetol.

[27] Nguyen CL, Pham NM, Binns CW, Van Duong D, Lee AH (2018). Prevalence of gestational diabetes mellitus in eastern and southeastern Asia: a systematic review and meta-analysis. J Diabetes Res.

[28] Solomon CG, Willett WC, Carey VJ (1997). A prospective study of pregravid determinants of gestational diabetes mellitus. JAMA.

[29] Retnakaran R, Connelly PW, Sermer M, Zinman B, Hanley AJG (2007). The impact of family history of diabetes on risk factors for gestational diabetes. Clin Endocrinol.

[30] Wu M, Wen J, Qin Y (2017). Familial history of diabetes is associated with poor glycaemic control in type 2 diabetics: a cross-sectional study. Sci Rep.

[31] Law JR, Stafford JM, D’Agostino RBJ (2015). Association of parental history of diabetes with cardiovascular disease risk factors in children with type 2 diabetes. J Diabetes Complications.

[32] Cheung JTK, Lau E, Tsui CCT (2022). Combined associations of family history and self-management with age at diagnosis and cardiometabolic risk in 86,931 patients with type 2 diabetes: joint Asia diabetes evaluation (JADE) Register from 11 countries. BMC Med.

[33] Katulanda P (2015). The influence of Family History of Diabetes on Disease Prevalence and Associated metabolic risk factors among Sri Lankan adults. Diabet Med.

